# Positive vaccine beliefs linked to reduced mental stress in healthcare professionals during COVID-19: a retrospective study

**DOI:** 10.3389/fpsyt.2024.1402194

**Published:** 2024-09-18

**Authors:** Yu-Yin Lin, Shih-Feng Cho, Yi-Ling Hsieh, Yun-Shiuan Chuang, Chia-En Hsu, Yun-Chen Liu, Chia-Chi Sung, Ya-Hsiu Huang, Wen Ku, Meng-Hsuan Hsieh, Ya-Chin Huang, Hung-Pin Tu, Chao-Ling Wang, Chi-Kung Ho

**Affiliations:** ^1^ Department of Preventive Medicine, Kaohsiung Medical University Hospital, Kaohsiung Medical University, Kaohsiung, Taiwan; ^2^ Department of Occupational Safety and Health, Kaohsiung Medical University Hospital, Kaohsiung Medical University, Kaohsiung, Taiwan; ^3^ Department of Occupational & Environmental Medicine, Kaohsiung Medical University Hospital, Kaohsiung Medical University, Kaohsiung, Taiwan; ^4^ Division of Hematology and Oncology, Department of Internal Medicine, Kaohsiung Medical University Hospital, Kaohsiung Medical University, Kaohsiung, Taiwan; ^5^ Department of Family Medicine, Kaohsiung Medical University Hospital, Kaohsiung Medical University, Kaohsiung, Taiwan; ^6^ Department of Preventive Medicine, Kaohsiung Municipal Ta-Tung Hospital, Kaohsiung Medical University, Kaohsiung, Taiwan; ^7^ Department of Public Health, College of Health Science, Kaohsiung Medical University, Kaohsiung, Taiwan

**Keywords:** coronavirus disease 2019, burnout, mood disorder, healthcare professionals, vaccine beliefs

## Abstract

**Background and aim:**

The COVID-19 pandemic has led to a significant adverse effect on the mental health of healthcare professionals. This study aims to assess the effects of the prolonged pandemic on burnout and mood disorders and to evaluate the influence of positive vaccination beliefs on these factors at a medical center during the extended COVID-19 pandemic.

**Methods:**

This retrospective study analyzed the results of an online questionnaire survey including burnout status and mood disorders from 2020 to 2022. The factors related to mood moderate/severe disorders and the impact of the positive vaccine belief were also explored.

**Results:**

The initial analysis revealed that healthcare professionals continued to experience significant levels of personal and work-related burnout, along with mood disorders. However, the scores and the percentage of moderate to severe burnout gradually decreased. Notably, the percentage of individuals with moderate to severe mood disorders also gradually declined (2020: 13.4%, 2021: 12.3%, 2022: 11.1%). The number of participants who need professional interventions decreased from 56.2% in 2020 to 45.9% in 2021, and 46% in 2022. Multivariate analysis revealed a positive vaccine belief was associated with a lower risk of moderate/severe mood disorders, with odd ratios (OR) and 95% confidence intervals (95% CI) of 0.38 (0.28 – 0.52) and 0.41 (0.30 – 0.52) in the 2021 and 2022 cohorts, respectively. Further investigation revealed that age over 50 was linked to a positive vaccine belief in 2021 and 2022. Within the 2022 cohort, working as nurses was identified as the independent factor associated with a less positive belief, with the OR and 95% CI of 0.49 (0.27 – 0.90).

**Conclusion:**

The findings of the present study suggest burnout and mood disorders are still significant during the pandemic. A positive vaccine belief may mitigate pandemic-related mental distress. Further interventions to enhance the belief combined with other supporting measures are important in a long fight against the pandemic.

## Introduction

Coronavirus disease 2019 (COVID-19), caused by the coronavirus SARS-CoV-2, is a pneumonia first detected in Wuhan, China, and then rapidly spread in 2020, leading to devastating consequences (1). To stop the rapid spread of COVID-19, several non-pharmacological measures were implanted, which helped limit the disease transmission. However, measures were variably implemented (2–4). The development and implementation of a large-scale vaccination project has had a profound impact and played a decisive role in the course of the pandemic. The COVID-19 vaccine stimulates the human body to recognize the virus, generate neutralizing antibodies, and establish a biological memory of the virus, providing sustainable immunity to recipients (5). In the clinical trials, the COVID-19 vaccine effectively protected the recipients from several complications and death with a satisfactory safety profile (6–10). In addition, the real-world investigation also supported that vaccination remained an important control strategy during waves of COVID-19 infection and the emergence of variants of the SARS-CoV-2 virus (11). Global COVID-19 vaccination has substantially altered the course of the pandemic, which reduces incidence, hospitalizations, and deaths, saving tens of millions of lives worldwide (12). In Taiwan, all healthcare professionals and related staff were prioritized and encouraged to receive COVID-19 vaccination since 2021.

During the pandemic period of COVID-19, healthcare professionals play a critical role in preventing community or nosocomial outbreaks, as well as clinical care of infected patients. However, the prolonged pandemic, serial measures to control COVID-19 dissemination, as well as associated clinical practice also put frontline healthcare professionals under unprecedented stress levels, leading to in prevalence of mental stress (13–15). A previous study has also shown that the COVID-19 pandemic exhibited a negative effect on the mental status of healthcare professionals. The percentages of severe burnout and mood disorder were significantly increased compared with the pre-pandemic era (16). Since the COVID-19 pandemic lasted for up to three years, longer than expected, the adverse impact on mental health is very important, conscious monitoring of mental status and providing of necessary healthcare support are mandatory.

The implantation of the large-scale vaccination project has significantly altered the course of the pandemic. With confirmed scientific rationale and rapid information dissemination during the pandemic, comprehensive investigations can provide deeper insights into its impact across various dimensions. It is important to understand whether the scientific success of vaccination can lead to a shift in perception of the pandemic, enhance belief in well-being, or reduce the risk of mental illness. Previous studies revealed COVID-19 vaccination was associated with improved psychological well-being and declined distress during the pandemic (17, 18). Another study also revealed people would feel safer and might relax their safe behaviors during vaccination campaigns regardless of the dynamics of the epidemic (19). Despite the scientific success of vaccination, vaccine hesitancy still exists among healthcare professionals, which is linked to a higher risk of anxiety and mental stress (20, 21). Factors such as younger age (<50 years) and being non-doctor healthcare personnel have been identified as risk factors for not having a COVID-19 vaccine or having it late (22). The dissemination of accurate scientific information about the vaccine and the disease can effectively help reduce vaccine hesitancy (23). Additionally, a greater intention to get vaccinated was associated with a lower risk of COVID-19-related burnout, and better mental resilience had a direct positive effect on the intention to get vaccinated (24). Furthermore, the belief benefit of COVID-19 vaccination would be positively associated with the number of vaccination injections (25). However, there are still limited studies exploring the impact of vaccination intention or positive beliefs on mental stress and burnout status among frontline healthcare professionals during the prolonged pandemic. The information regarding the effect of the prolonged pandemic on burnout status and mood disorder remained scarce.

Herein, we conducted a retrospective study by analyzing online survey data from frontline healthcare professionals from 2020 to 2022 to elucidate the impact of the prolonged pandemic on burnout status and mood disorders. This study also investigated the effect of positive vaccination beliefs on burnout status and mood disorders, and the factors related to positive vaccine beliefs in a medical center during the COVID-19 pandemic.

## Methods

### Study design

**Figure 1:**
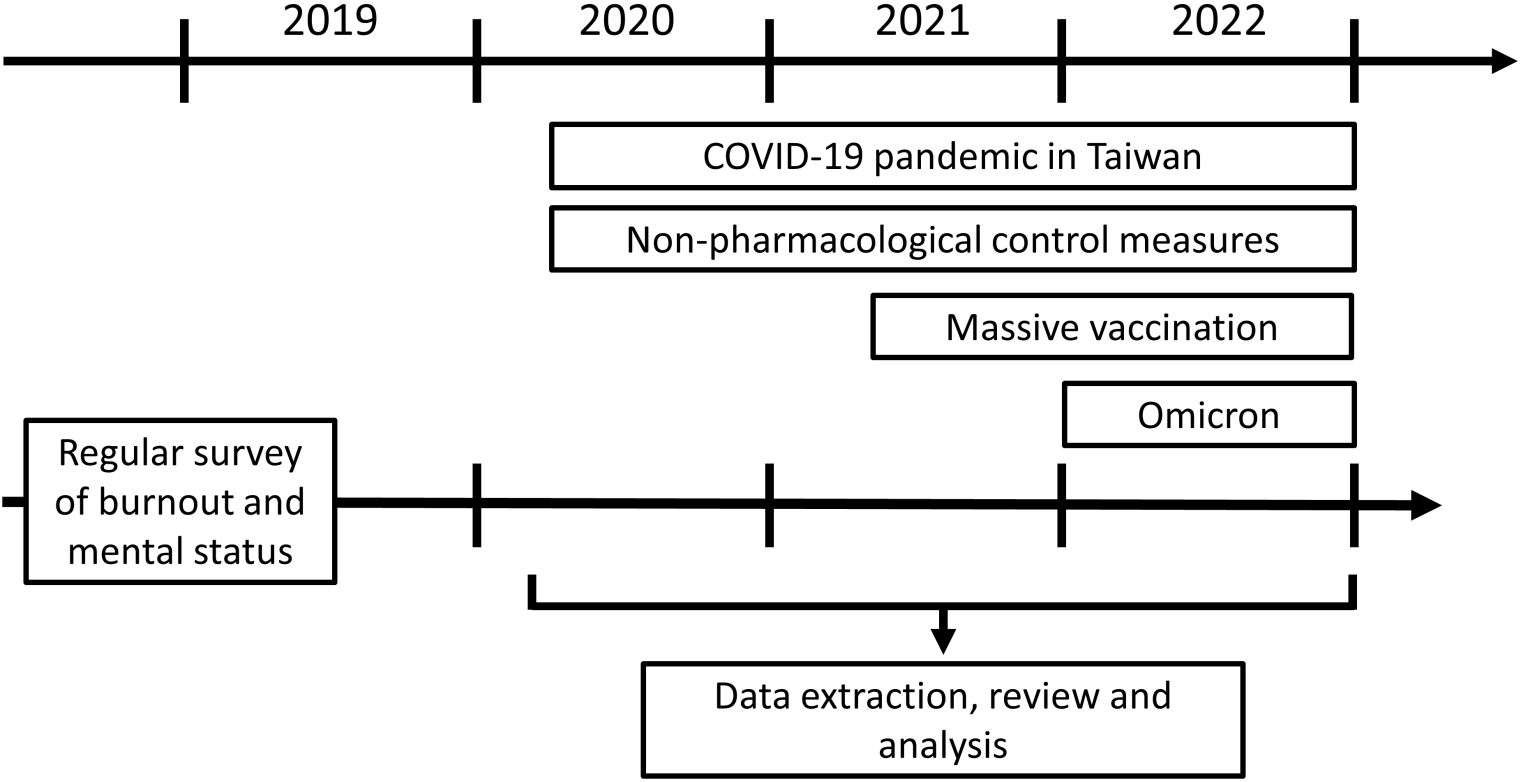
The important time points of the COVID pandemic in Taiwan. This retrospective analysis reviewed investigated the questionnaire data which were answered in some main periods of the COVID-19 pandemic. The initial surge of COVID-19 cases in Taiwan occurred in March 2020, leading to the implementation of a series of non-pharmacological control measures. Starting from March 2021, the massive vaccination project targeting healthcare professionals was initiated. Throughout 2021, various strains of the SARS-CoV virus were detected. In 2022, a major milestone was the identification of the Omicron variant, accompanied by an obvious surge in case numbers.

This study aims to investigate the occupational burnout index and mental illness among hospital employees during the COVID-19 pandemic, as well as the impact of medical interventions such as massive vaccination. All the data for this study were retrospectively collected from the results of an online questionnaire, which is part of the annual health exam for adult employees (aged ≥ 20 years) at Kaohsiung Medical University Hospital, a medical center with approximately 4200 employees and a major COVID-19 first-line hospital in southern Taiwan. This questionnaire survey had been conducted regularly before the pandemic. The evaluation of the questionnaire includes the assessment of personal burnout, work-related burnout, and mood disorders. Throughout the COVID-19 pandemic, the survey remained ongoing for the implantation of supporting measures. For the present study, The results of questionnaires from three periods between 2020 and 2022 were extracted for analysis (**Figure 1**).

The details of how personal and work-related burnout, as well as mood disorders, were assessed were described in the previous study (16). In brief, the degree of severity of personal burnout was determined by the average of six questions with five scores (0, 25, 50, 75, and 100), with scores less than 50 (< 50), 50 to 70, and more than 70 (> 70) representing no/mild, moderate, and severe personal burnout, respectively. The severity of work-related burnout was determined by the average of seven questions with five scores (0, 25, 50, 75, and 100), with scores of less than 45 (< 45), 45 to 60, and more than 60 (> 60) indicating no/mild, moderate, and severe work-related burnout, respectively (26) (**Supplementary Figure 1**). The mood disorder was investigated using a 5-item brief symptom rating scale (BSRS-5) (27, 28). The mood disorder was defined by the presence of psychological symptoms, including anxiety, depression, hostility, interpersonal sensitivity, and additional issues, such as difficulty falling asleep in the past week (28). The participants who reported a total score above 14 (≥ 15), or a score of more than 1 on the additional suicide survey item were considered a severe mood disorder. Scores between 10 and 14, 6 and 9, and 0 and 5 represent moderate, mild, and no/minimal mood disorders, respectively. Since 2021, a massive vaccination program was initiated, and the number of vaccinated healthcare professionals increased significantly from mid-2021 after an episode of the COVID-19 case surge. In 2021 and 2022, a new question was added to the questionnaire, the participants voluntarily answered whether vaccination was “helpful (scores 4 and 5)” or “not quite helpful (scores 1 to 3)” in alleviating their burnout or mood disorders. Specifically, scores of 1, 2, and 3 corresponded to ‘totally not helpful at all,’ ‘not helpful,’ and ‘neutral/not sure,’ respectively. Scores of 4 and 5 corresponded to ‘helpful’ and ‘very helpful,’ respectively. Participants who answered 4 or 5 were categorized as belonging to the group with a positive belief in the vaccine’s efficacy. To evaluate the impact of vaccination belief, the data from October 2021 was extracted, at that time, nearly all the healthcare professionals (> 99%) at Kaohsiung Medical University Hospital had received at least one dose of the COVID-19 vaccine.

### Human ethics and consent to participate declarations

This research was conducted in accordance with the Declaration of Helsinki. The protocol was approved by the Institutional Review Board of Kaohsiung Medical University Hospital (Approval Number: KMUHIRB-E(I)-20200292). The participating employees voluntarily provided consent and then completed the questionnaire.

### Statistics

All eligible data was incorporated in the analysis for this study. Descriptive statistics were utilized to provide a summary of the findings. Continuous variables, including the number, mean values, standard deviation, and median, were presented (shown as means ± SDs). The independent two-sample t-test or ANOVA test (followed by LSD/S-N-K *post-hoc* tests) was utilized to examine differences in continuous variables. Categorical variables, including the number and percentages of subjects in each class, were presented, and their frequencies were assessed by the Chi-squared test (χ^2^ test). The evaluation of correlation was carried out by Pearson correlation analysis. The univariate and multivariate logistic regression analyses were conducted to elucidate the relative risk associated with each parameter. The odds ratios (ORs) and 95% confidence intervals (CIs) were also calculated. A *P*-value less than 0.05 indicated statistical significance.

## Results

### The issues of burnout and mood disorder remained significant but may gradually improve

**Figure 2:**
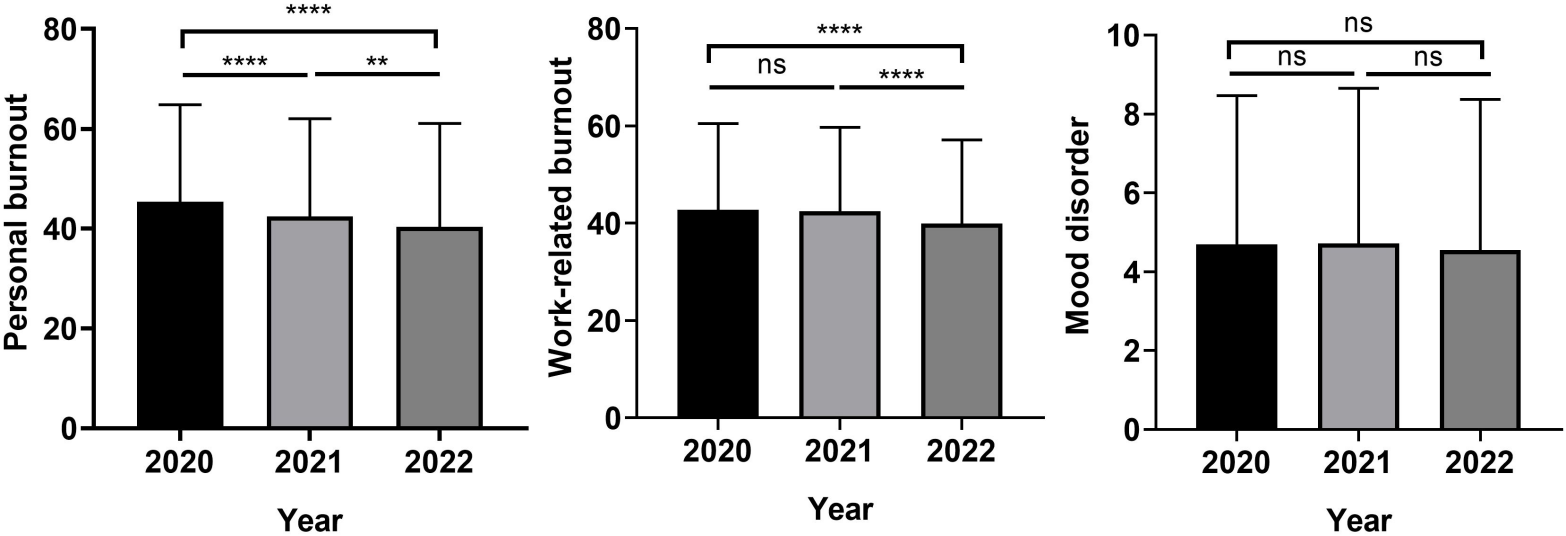
Personal burnout, work-related burnout, and mood disorder scores during the COVID-19 pandemic. (**P <0.01; ****P< 0.0001; ns, not significant).

In the study period of 2020, 2021, and 2022, there were approximately 2000. 1500, and 1800 employees with mean ages of 40.4, 42.5, and 43.5 years, respectively, completed the survey. The result revealed that the score of personal, and work-related burnout remained high in 2021 and 2022 compared with 2020. Importantly, there was a trend of decreasing mean scores of personal burnout in 2021 (42.30 ± 19.58) and 2022 (40.31 ± 20.77), which were statistically lower than in 2020 (45.38 ± 19.44). Similarly, the mean of work-related burnout scores were significantly lower in 2022 (39.88 ± 17.25), lower than in 2020 (42.78 ± 17.68) and 2021 (42.46 ± 17.24). No significant changes in mood disorder scores (2020: 4.69 ± 3.78, 2021: 4.71 ± 3.94, 2022: 4.55 ± 3.82) were observed (**Figure 2**). In addition, the percentage of the participants who need professional interventional, like psychological assistance, decreased from 56.2% in 2020 to 45.9% in 2021, and 46% in 2022 (P < 0.001). Regarding the type of assistance, more rest, perquisite, and less loading remained the most desired assistance from the hospital in 2021 (81%, 76.1%, 66.3%) and 2022 (78.5%, 72.5%, 63.2%).

With respect to the severity of distress, we observed the percentage of moderate or severe burnout, including personal and work-related, has decreased gradually since 2021. Additionally, from 2020 to 2022, the percentage of moderate/severe mood also decreased, from 13.4% in 2020 to 11.1% in 2022, while the percentage of mild mood disorder increased, from 18.6% in 2020 to 23.2% in 2022 (**Table 1**).

**Table 1:** The detailed summary of person burnout, work-related burnout, and mood disorder in the study group during the COVID pandemic (2020-2022).

		2020 (n= 2029)	2021 (n= 1489)	2022 (n= 1753)	*P*-value
Personal	Mild	1128 (55.6)	906 (60.8)	1084 (61.8)	< 0.001
	Moderate	645 (31.8)	429 (28.8)	503 (28.7)	
	Severe	256 (12.6)	154 (10.3)	166 (9.5)	
		2020 (n= 2029)	2021 (n= 1488)	2022 (n= 1753)	
Work	Mild	1106 (54.5)	828 (55.1)	1052 (60.0)	0.008
	Moderate	612 (30.2)	445 (29.6)	485 (27.7)	
	Severe	311 (15.3)	215 (14.3)	216 (12.3)	
		2020 (n= 2029)	2021 (n= 1488)	2022 (n= 1809)	
Mood	None	1379 (68.0)	970 (65.2)	1188 (65.7)	0.003
	Mild	379 (18.6)	335 (22.5)	420 (23.2)	
	Moderate	227 (11.2)	159 (10.7)	181 (10.0)	
	Severe	44 (2.2)	24 (1.6)	20 (1.1)	

Chi-square analysis.

### The participants with vaccination beliefs have lower burnout and mood disorder scores

**Figure 3:**
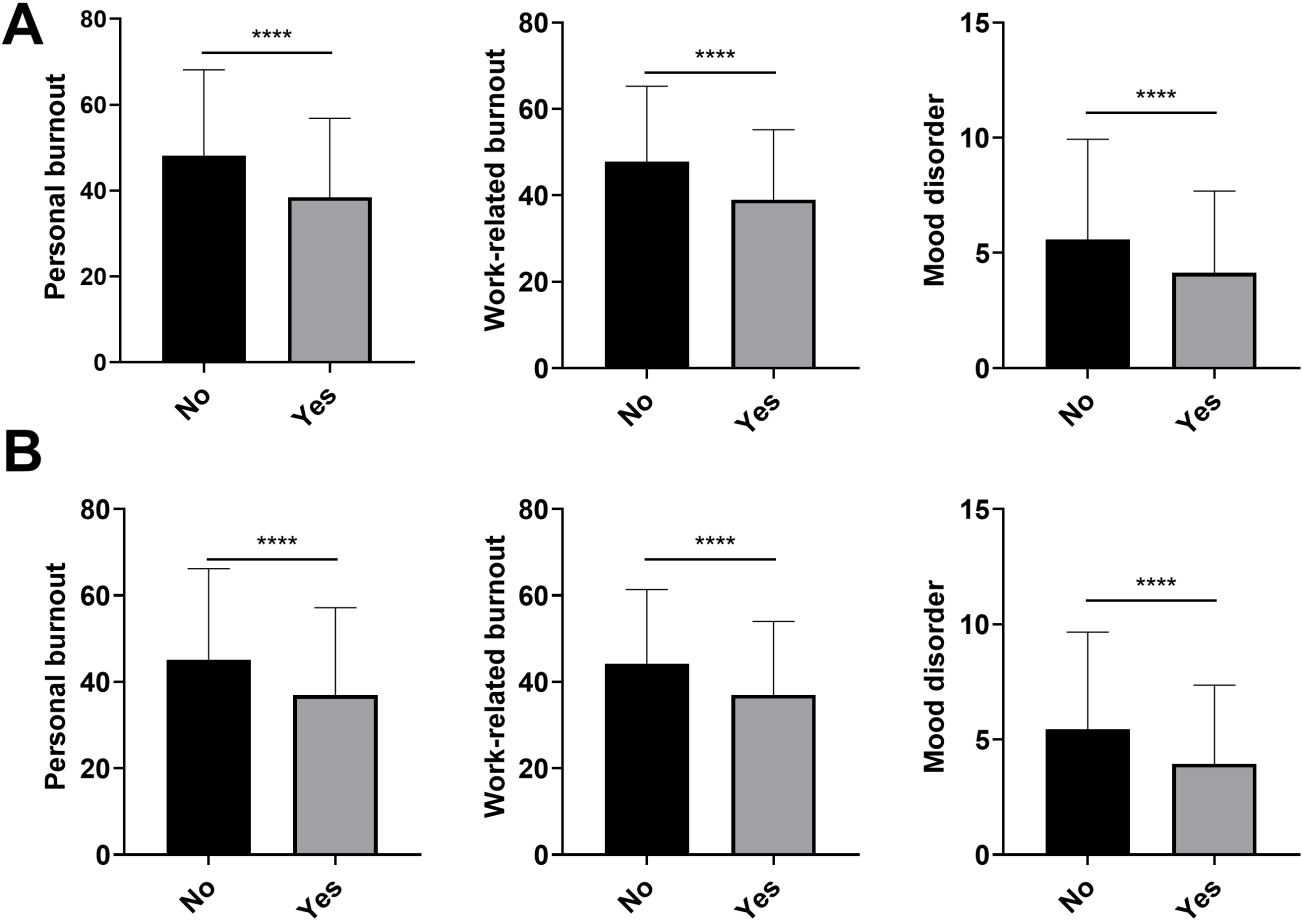
Personal burnout, work-related burnout, and mood disorder scores in participants with different vaccine beliefs (Positive belief: Yes vs No) in the 2021 **(A)** and 2022 cohorts **(B)**. (****P< 0.0001).

Massive vaccination plays a key factor in shifting the trajectory and mitigating the devastating consequences of COVID-19, we next elucidate if this intervention is associated with a decreased risk of burnout or mood disorder. The participants were divided into two groups based on their self-reported results, indicating whether they had a positive belief in the vaccine’s efficacy or not. Based on the analysis, the percentages of participants who reported finding positive vaccine belief were 66.3% and 59.7% in 2021 and 2022, respectively. Notably, the percentages of moderate and severe personal or work-related, as well as mood disorders were significantly lower in the group with positive vaccine belief (**Table 2**). The score of personal burnout, work-related burnout, and mood disorder were significantly lower in the positive vaccine belief group regardless of 2021 (personal: 48.14 ± 19.98 vs 38.48 ± 18.35, work: 47.77 ± 17.49 vs 39.00 ± 16.18, mood: 5.57 ± 4.36 vs 4.15 ± 3.54) or 2022 (personal: 45.17 ± 21.06 vs 36.96 ± 20.20, work: 44.24 ± 17.14 vs 36.97 ± 17.02, mood: 5.45 ± 4.21 vs 3.94 ± 3.41) (**Figure 3**).

**Table 2:** The summary of the association between burnout, mood disorder, and the positive vaccination belief (Yes vs No).

Year		2021 n=1488		2022 n=1753	
Belief (No or Yes)		No (n=587)	Yes (n=901)	No (n=706)	Yes (n=1047)
Personal	Mild	274 (46.7)	632 (70.2)	370 (52.4)	714 (68.2)
	Moderate	217 (37.0)	211 (23.4)	235 (33.3)	268 (25.6)
	Severe	96 (16.3)	58 (6.4)	101 (14.3)	65 (6.2)
*P*-value		< 0.001		< 0.001	
		n=1487^#^		n=1753	
Belief (No or Yes)		No (n=587)	Yes (n=900)	No (n=706)	Yes (n=1047)
Work	Mild	241 (41.0)	587 (65.2)	341 (48.3)	711 (67.9)
	Moderate	227 (38.7)	217 (24.1)	247 (35.0)	238 (22.7)
	Severe	119 (20.3)	96 (10.7)	118 (16.7)	98 (9.4)
*P*-value		< 0.001		< 0.001	
		n=1488		n=1809	
Belief (No or Yes)		No (n=587)	Yes (n=901)	No (n=725)	Yes (n=1084)
Mood	None/mild	475 (80.9)	830 (92.1)	599 (82.6)	1009 (93.1)
	Moderate/ severe	112 (19.1)	71 (7.9)	126 (17.4)	75 (6.9)
*P*-value		< 0.001		< 0.001	

^#^One person didn’t answer the vaccine belief question.

Chi-square analysis.

We next investigated the factors related to moderate/severe mood disorder in 2021 and 2022. Unlike the data we previously published, which revealed job titles like nurse and working in the emergent department were related to higher risk (16), the analysis for 2021 and 2022 didn’t identify independent adverse factors for poorer mood status. The univariate analysis indicated that age over 50 and having a positive belief in vaccines would be associated with a reduced risk of experiencing moderate/severe mood disorders. In terms of multivariate analysis, the findings suggested that a positive belief in vaccines was the sole independent factor linked to a reduced risk of moderate/severe mood disorders, with ORs and 95% CIs of 0.38 (0.28 – 0.52) and 0.41 (0.30 – 0.52) in 2021 and 2022 (both P < 0.001), respectively (**Table 3**, details in **Supplementary Tables 1, 2**).

**Table 3:** Investigation of the factors related to moderate/severe mood disorder in 2021 and 2022.

	Univariate		Multivariate	
Variables	Crude OR 95%CI	*P*-value	Adjusted OR 95%CI	*P*-value
2021				
Age > 50 years	0.60 (0.34 -1.07)	0.083	0.78 (0.43 – 1.39)	0.398
Positive belief	0.36 (0.26 – 0.50)	< 0.001	0.38 (0.28 – 0.52)	< 0.001
2022				
Age > 50 years	0.53 (0.29 -0.97)	0.040	0.68 (0.37 – 1.27)	0.224
Positive belief	0.35 (0.26 – 0.48)	< 0.001	0.41 (0.30 – 0.52)	< 0.001

The variables with P-value < 0.1 in univariate analysis were included in the multivariate analysis.

### Exploration of factors related to a positive vaccine belief

Since a positive belief in vaccination is associated with a lower risk of poorer mood status, we further evaluate which relevant factors. The results revealed that older age (> 50 years), male gender, and non-frontline job title or working place were associated with a higher percentage of a positive vaccine belief. On the other hand, younger staff (aged 20 to 30 years), female gender, nurses, patient contact, and working in the intensive care units or isolation wards showed a significantly lower percentage of positive vaccine belief (**Table 4**).

**Table 4:** The summary of the positive vaccine belief (Yes vs No) in different ages, genders, job titles, patient contact, and working areas.

Age (years)	2021 (n=1483)		2022 (n=1734)	
	No (n=586)	Yes (n=897)	No (n=696)	Yes (n=1038)
21 - 30	102 (50.0)	102 (50.0)	97 (53.9)	83 (46.1)
31 - 40	172 (40.9)	249 (59.1)	203 (41.4)	287 (58.6)
41 - 50	233 (43.5)	303 (56.5)	289 (44.9)	354 (55.1)
> 50	79 (24.5)	243 (75.5)	107 (25.4)	314 (74.6)
*P*-value	< 0.001		< 0.001	
Gender	2021 (n=1488)		2022 (n=1809)	
	No (n=587)	Yes (n=901)	No (n=725)	Yes (n=1084)
Male	55 (30.6)	125 (69.4)	77 (31.7)	166 (68.3)
Female	532 (40.2)	776 (58.8)	648 (41.4)	918 (58.6)
*P*-value	0.005		0.031	
Job titles	2021 (n=1414)		2022 (n=1734)	
	No (n=563)	Yes (n=851)	No (n=696)	Yes (n=1038)
Physicians	11 (33.3)	22 (66.7)	19 (25.7)	55 (74.3)
Nurses	393 (45.6)	468 (54.4)	446 (47.2)	499 (52.8)
Medical staffs	56 (28.4)	141 (71.6)	71 (29.0)	174 (71.0)
Technicians	12 (31.6)	26 (68.4)	11 (28.2)	28 (71.8)
Administration	91 (31.8)	194 (68.2)	149 (34.6)	282 (65.4)
*P*-value	< 0.001		< 0.001	
Patient contact	2021 (n=1485)		2022 (n=1794)	
	No (n=587)	Yes (n=898)	No (n=720)	Yes (n=1074)
Yes	486 (42.0)	672 (58.0)	593 (43.0)	785 (57.0)
No	101(30.9)	226 (69.1)	127 (30.5)	289 (69.5)
*P*-value	< 0.001		< 0.001	
Working sites	2021 (n=1445)		2022 (n=1790)	
	No (n=587)	Yes (n=858)	No (n=719)	Yes (n=1071)
ER	42 (44.2)	53 (55.8)	50 (46.7)	57 (53.3)
ICU/isolation	95 (51.9)	88 (48.1)	107 (51.2)	102 (48.8)
General ward	211 (44.7)	261 (55.3)	241 (45.1)	293 (54.9)
OPD/exam rooms	83 (34.9)	155 (65.1)	108 (33.2)	217 (66.8)
RnC/PS/P	29 (37.2)	49 (62.8)	31 (37.3)	52 (62.7)
Administrative	50 (27.0)	135 (73.0)	63 (30.3)	145 (69.7)
Others	77 (32.3)	157 (67.7)	113 (36.7)	205 (63.3)
*P*-value	< 0.001		< 0.001	

ER, emergent room; ICU, intensive care unit; OPD, outpatient department; RnC/PS/P, Registration and Cashier/patient service/pharmacy.

Chi-square analysis.

Further evaluation of the factors related to vaccine belief was performed. In the 2021 cohort, the univariate analysis showed female and patient contact was associated with a less positive vaccine belief. Senior staff (age > 50 years), and working in the non-frontline area were related to a more positive vaccine belief. The results of multivariate analysis revealed older age (> 50 years) and working in the administrative area were independent factors for a more positive vaccine belief. In the 2022 cohort, the univariate analysis showed female gender and patient contact were associated with a less positive vaccine belief. In multivariate analysis, age between 31 and 40 or > 50 years was related to a more positive vaccine belief, however, we identified that the nurse title was the independent factor for a less positive belief, with ORs and 95% CIs of 0.49 (0.27 – 0.90) (**Table 5**, details in **Supplementary Tables 3, 4**).

**Table 5:** Investigation of the factors related to positive vaccine belief in 2021 and 2022.

	Univariate		Multivariate	
Variables	Crude OR 95%CI	*P*-value	Adjusted OR 95%CI	*P*-value
2021				
Female	0.64 (0.46 – 0.90)	0.010	0.74 (0.52 – 1.06)	0.100
Age > 50 years	3.08 (2.12 – 4.47)	<0.001	2.67 (1.81 – 3.93)	<0.001
Patient contact: Yes	0.62 (0.48 – 0.80)	<0.001	1.06 (0.74 – 1.51)	0.751
Working space/area				
Administrative area	2.14 (1.27 – 3.59)	0.004	2.03 (1.12 – 3.71)	0.020
Others^#^	1.62 (0.99 – 2.63)	0.054	1.06 (0.93 – 2.57)	0.092
2022				
Female	0.66 (0.49 – 0.88)	0.004	0.95 (0.67 – 1.36)	0.780
Age (years)				
31 - 40	1.65 (1.17 – 2.22)	0.004	1.49 (1.05 - 2.13)	0.025
41 - 50	1.43 (1.03 – 1.99)	0.034	1.24 (0.88 – 1.75)	0.214
> 50	3.34 (2.38 – 4.95)	<0.001	2.77 (1.88 – 4.08)	<0.001
Nurses	0.38 (0.23 – 0.66)	0.001	0.49 (0.27 – 0.90)	0.022
Patient contact: Yes	0.58 (0.46 – 0.74)	<0.001	0.85 (0.61 – 1.18)	0.331
Working area				
OPD/exam rooms	1.76 (1.13 – 2.75)	0.012	1.50 (0.94 – 2.39)	0.087
Administrative area	2.02 (1.25 – 3.27)	0.004	1.52 (0.85 – 2.73)	0.160
Others^#^	1.51 (0.97 – 2.35)	0.067	1.20 (0.75 – 1.93)	0.452

The variables with P-value < 0.1 in univariate analysis were included in the multivariate analysis.

OPD, outpatient department.

^#^working site other than ER, ICU, OPD/exam room, RnC/PS/P, isolation ward, general ward, and administrative area.

## Discussion

The COVID-19 pandemic has significantly devastated the healthcare systems and impacted mental health globally. Despite various effective prevention measures and pharmacologic interventions like vaccines or antiviral agents, the evolution and emergence of novel virus variants have prolonged the pandemic beyond initial expectations (29–31). Accumulating evidence indicates a negative effect on mental health status, characterized by the onset of psychological symptoms such as depression, panic disorder, insomnia, and anxiety, particularly among frontline healthcare workers over time (32–35). In this study, we investigated burnout status, mood disorder, and the effect of the massive vaccination project on healthcare professionals. The first main finding reveals that the condition of burnout and mood disorders remains a significant issue during the COVID-19 pandemic.

Of note, we observed the most significant impact of the COVID-19 pandemic in 2020 compared to 2021 and 2022. The percentage of moderate/severe personal and work-related burnout, as well as mood disorders, gradually decreased. This decline can be attributed to several factors. For example, timely psychological support and adequate personal protective equipment may alleviate the stress experienced by frontline healthcare professionals. Moreover, the rapid dissemination of positive information, including the scientific success of the vaccine, improved crisis communication, and the implementation of large-scale vaccination campaigns, also played a role in mitigating the escalation of pandemic-related stress, potentially even partially overcoming it (36, 37).

Another critical aspect evaluated in this study is the effect of positive vaccine belief. The initial results of the multivariate analyses revealed no independent risk for moderate to severe mood disorders in the 2021 and 2022 cohorts, suggesting the overwhelming impact of the prolonged COVID-19 pandemic on mood status, regardless of differences in age, gender, patient contact, job title, or work sites. However, a positive vaccine belief was revealed to be linked to a lower risk of moderate/severe mood disorder in these cohorts. In addition, the participants with a positive vaccine belief reported lower burnout and mood disorder scores. This result was concordant with the previous study which revealed that vaccination may alleviate COVID-19-related psychological distress (38), especially in individuals with more positive beliefs.

The present also explored the factors related to a positive vaccine belief. The senior staff aged 50 years and older was linked to a more positive belief in multivariate analysis and a lower risk of mood disorder in univariate analysis. One of the possible explanations would be the previous experience of the outbreak of SARS in their early career, along with psychological maturity, making them more mentally prepared when facing the COVID-19 pandemic (39, 40). In addition, this group is less likely to have vaccine hesitancy than the younger age group (41, 42). However, in the 2022 cohort, we found that the work as nurses was the independent factor of a less positive belief. The underlying reason for this finding could be attributed to the significantly increased likelihood of direct patient contact, as well as the longer time in direct patient care. Given working as a nurse has been associated with a higher incidence of psychological distress (43, 44), it is worth investigating whether this distress could potentially lead to less positive beliefs. Moreover, this finding also highlights the importance of supportive measures during the prolonged COVID-19 pandemic since this subgroup is more likely to benefit from these interventions (45).

The study may have some strengths and limitations. First, it analyzed data from a large cohort of participants, representing a wide range of hospital job categories. The research also compared the mood and burnout status of healthcare professionals with varying vaccine beliefs and examined data across different stages of pandemic prevention over three consecutive years. Such longitudinal studies are relatively uncommon in COVID-19 research. However, there are some limitations. First, it is a retrospective study by analyzing self-reported data, there may exist confounding factors leading to potential biases. Second, the analysis may be limited in its ability to explore job categories with fewer participants. Additionally, as the study was conducted at a single institution, the findings may lack broader generalizability. Including data from multiple healthcare centers in future studies could enhance the comprehensiveness and representativeness of the findings. Lastly, a longer observational period may provide more detailed insights, given the ongoing development of new virus variants and the prevalence of new or re-infected COVID-19 cases worldwide.

In summary, this study demonstrated that burnout and mood disorders remain critical issues in healthcare professionals during the COVID-19 pandemic. With the implantation of effective supportive measures, these conditions may improve gradually. In addition, we found that a more positive vaccine belief may be associated with a lower risk of significant burnout and mood disorder. The factors related to more or less positive vaccine beliefs were also identified in this study. In conclusion, the findings of this study highlight the importance of supportive measures, the dissemination of timely and accurate information, improved critical communication, and education to foster a positive culture. These efforts may further strengthen positive beliefs and reduce pandemic-related stress.

## Data Availability

The original contributions presented in the study are included in the article/**Supplementary Material**. Further inquiries can be directed to the corresponding author.
