# Supplementary material for: Positive vaccine beliefs linked to reduced mental stress in healthcare professionals during COVID-19: a retrospective study
Source: Front Psychiatry. 2024 Sep 18;15:1402194. doi: 10.3389/fpsyt.2024.1402194 (PMC11445048; doi:10.3389/fpsyt.2024.1402194)
Supplement: Supplementary file 1 [file DataSheet1.pdf]

Supplemental Materials

Positive Vaccine Beliefs Linked to Reduced Mental Stress in Healthcare Professionals During COVID-19: A Retrospective Study

Supplemental Figure 1. The burnout questionnaire for this study.

Personal Burnout Questions

1. How often do you feel tired?
(4) Always (3) Often (2) Sometimes (1) Seldom (0) Never/rarely.
2. How often are you physically exhausted?
(4) Always (3) Often (2) Sometimes (1) Seldom (0) Never/rarely.
3. How often are you emotionally exhausted?
(4) Always (3) Often (2) Sometimes (1) Seldom (0) Never/rarely.
4. How often do you think: "I can't take it anymore"?
(4) Always (3) Often (2) Sometimes (1) Seldom (0) Never/rarely.
5. How often do you feel worn out?
(4) Always (3) Often (2) Sometimes (1) Seldom (0) Never/rarely.
6. How often do you feel weak and susceptible to illness?
(4) Always (3) Often (2) Sometimes (1) Seldom (0) Never/rarely.

(4) Always: 100. (3) Often: 75. (2) Sometimes: 50. (1)Seldom: 25. (0) Never/rarely: 0.
The score of personal burnout is the average score of the above 6 questions (1-6).

Work-related Burnout Questions

7. Is your work emotionally exhausting?
(4) To a very high degree (3) To a high degree (2) Somewhat (1) To a low degree (0) To a very low degree.
8. Do you feel burnt out because of your work?
(4)To a very high degree (3)To a high degree (2)Somewhat (1)To a low degree (0) To a very low degree.
9. Does your work frustrate you?
(4) To a very high degree (3) To a high degree (2) Somewhat (1) To a low degree (0) To a very low degree.
10. Do you feel worn out at the end of the working day?
(4) Always (3) Often (2) Sometimes (1) Seldom (0) Never/rarely.
11. Are you exhausted in the morning at the thought of another day at work?
(4) Always (3) Often (2) Sometimes (1) Seldom (0) Never/rarely.
12. Do you feel that every working hour is tiring for you?
(4) Always (3) Often (2) Sometimes (1) Seldom (0) Never/rarely.
13#. Do you have enough energy for family and friends during your leisure time?
(4) Always (3) Often (2) Sometimes (1) Seldom (0) Never/rarely.

(4) Always: 100. (3) Often: 75. (2) Sometimes: 50. (1)Seldom: 25. (0) Never/rarely: 0.
The score of work-related burnout is the average score of the above 7 questions (7-13).
Reversed score for the last question.

Reference:

1. Yeh W.Y., Cheng Y., Chen C.J., Hu P.Y., Kristensen T.S. Psychometric properties of the Chinese version of Copenhagen burnout inventory among employees in two companies in Taiwan. Int. J. Behav. Med. 2007;14:126–133.

Supplemental Table 1. Investigation of the factors related to moderate/severe mood disorder in 2021 (Detail).

Variables	Crude OR 95%CI	*P*-value	Adjusted OR 95%CI	*P*-value
Gender				
Male	1 (Ref.)			
Female	1.05 (0.66-1.68)	0.835		
Age (years)				
21-30	1 (Ref.)		1 (Ref.)	
31-40	1.07 (0.65 – 1.76)	0.784	1.18 (0.71 – 1.95)	0.530
41-50	1.06 (0.66 – 1.72)	0.805	1.13 (0.70 – 1.85)	0.613
> 50	0.60 (0.34 -1.07)	0.083	0.78 (0.43 – 1.39)	0.398
Title				
Physician	1 (Ref.)			
Nurse	0.84 (0.32 - 2.21)	0.720		
Medical staff	0.49 (0.17 – 1.46)	0.202		
Technician	0.48 (0.11 – 2.18)	0.342		
Administration	0.89 (0.32 – 2.44)	0.817		
Patient contact				
No	1 (Ref.)			
Yes	1.05 (0.72 -1.53)	0.805		
Working area				
ER	1 (Ref.)			
ICU/isolation wards	1.18 (0.59 – 2.34)	0.636		
General wards	0.75 (0.40 -1.41)	0.368		
OPD/exam rooms	0.71 (0.35 – 1.43)	0.336		
RnC/PS/P	1.05 (0.46 – 2.43)	0.906		
Administrative area	0.70 (0.34 – 1.46)	0.343		
Others	0.72 (0.38 – 1.45)	0.363		
Vaccine belief				
No	1 (Ref.)		1 (Ref.)	
Yes	0.36 (0.26 – 0.50)	< 0.001	0.38 (0.28 – 0.52)	< 0.001

The parameters with $p < 0.1$ in univariate were included in the multivariate analysis. ER, emergency room; ICU, intensive care unit; OPD, outpatient department; RnC/PS/P, registration and cashier/patient service/pharmacy.

Supplemental Table 2. Investigation of the factors related to moderate/severe mood disorder in 2022 (Detail).

Variables	Crude OR 95%CI	*P*-value	Adjusted OR 95%CI	*P*-value
Gender				
Male	1 (Ref.)			
Female	0.95 (0.62-1.47)	0.826		
Age (years)				
21-30	1 (Ref.)		1 (Ref.)	
31-40	1.16 (0.68 – 1.98)	0.590	1.23 (0.75 – 2.23)	0.347
41-50	1.20 (0.72 – 2.02)	0.486	1.30 (0.77 – 2.20)	0.320
> 50	0.53 (0.29 -0.97)	0.040	0.68 (0.37 – 1.27)	0.224
Category Group				
Physician	1 (Ref.)			
Nurse	1.34 (0.60 - 2.98)	0.475		
Medical staff	0.81 (0.32 – 1.97)	0.639		
Technician	1.09 (0.30 – 3.99)	0.892		
Administration	1.14 (0.49 – 2.64)	0.753		
Patient contact				
No	1 (Ref.)			
Yes	1.12 (0.79 -1.60)	0.522		
Working area				
ER	1 (Ref.)			
ICU/isolation wards	1.46 (0.70 – 3.05)	0.310		
General wards	0.98 (0.49 -1.95)	0.958		
OPD/exam rooms	1.05 (0.52 – 2.16)	0.887		
RnC/PS/P	1.47 (0.62 – 3.53)	0.383		
Administrative area	0.93 (0.43 – 2.02)	0.851		
Others	1.16 (0.57 – 2.36)	0.683		
Vaccine belief				
No	1 (Ref.)		1 (Ref.)	
Yes	0.35 (0.26 – 0.48)	< 0.001	0.41 (0.30 – 0.52)	< 0.001

The parameters with $p < 0.1$ in univariate were included in the multivariate analysis. ER, emergency room; ICU, intensive care unit; OPD, outpatient department; RnC/PS/P, registration and cashier/patient service/pharmacy.

Supplemental Table 3. Investigation of the factors related to positive vaccine belief in 2021 (Detail).

Variables	Crude OR 95%CI	*P*-value	Adjusted OR 95%CI	*P*-value
Gender group				
Male	1 (Ref.)		1 (Ref.)	
Female	0.64 (0.46 – 0.90)	0.010	0.74 (0.52 – 1.06)	0.100
Age subgroup (years)				
21-30	1 (Ref.)		1 (Ref.)	
31-40	1.45 (1.03 – 2.03)	0.031	1.38 (0.98 - 1.95)	0.062
41-50	1.30 (0.94 - 1.80)	0.111	1.23 (0.88 – 1.72)	0.219
> 50	3.08 (2.12 – 4.47)	<0.001	2.67 (1.81 – 3.93)	<0.001
Category Group				
Physician	1 (Ref.)			
Nurse	0.60 (0.28 – 1.24)	0.167		
Medical staff	1.26 (0.57 – 2.77)	0.566		
Technician	1.08 (0.40 – 2.93)	0.875		
Administration	1.07 (0.49 – 2.29)	0.870		
Patient contact				
No	1 (Ref.)		1 (Ref.)	
Yes	0.62 (0.48 – 0.80)	<0.001	1.06 (0.74 – 1.51)	0.751
Working space/area				
ER	1 (Ref.)			
ICU/isolation wards	0.73 (0.45 – 1.21)	0.224	0.80 (0.48 – 1.34)	0.400
General wards	0.98 (0.63 – 1.53)	0.980	1.05 (0.67 – 1.66)	0.822
OPD/exam rooms	1.48 (0.91 – 2.40)	0.113	1.43 (0.87 - 2.34)	0.156
RnC/PS/P	1.34 (0.73 – 2.47)	0.350	1.21 (0.65 – 2.25)	0.557
Administrative area	2.14 (1.27 – 3.59)	0.004	2.03 (1.12 – 3.71)	0.020
Others	1.62 (0.99 – 2.63)	0.054	1.06 (0.93 – 2.57)	0.092

The parameters with $p < 0.1$ in univariate were included in the multivariate analysis. ER, emergency room; ICU, intensive care unit; OPD, outpatient department; RnC/PS/P, registration and cashier/patient service/pharmacy.

Supplemental Table 4. Investigation of the factors related to positive vaccine belief in 2022 (Detail).

Variables	Crude OR 95%CI	*P*-value	Adjusted OR 95%CI	*P*-value
Gender group				
Male	1 (Ref.)		1 (Ref.)	
Female	0.66 (0.49 – 0.88)	0.004	0.95 (0.67 – 1.36)	0.78
Age subgroup (years)				
21-30	1 (Ref.)		1 (Ref.)	
31-40	1.65 (1.17 – 2.22)	0.004	1.49 (1.05-2.13)	0.025
41-50	1.43 (1.03 – 1.99)	0.034	1.24 (0.88 – 1.75)	0.214
> 50	3.34 (2.38 – 4.95)	<0.001	2.77 (1.88 – 4.08)	<0.001
Category Group				
Physician	1 (Ref.)		1 (Ref.)	
Nurse	0.38 (0.23 – 0.66)	0.001	0.49 (0.27 – 0.90)	0.022
Medical staff	0.85 (0.47 – 1.53)	0.580	0.90 (0.47 – 1.71)	0.746
Technician	0.88 (0.37 – 2.10)	0.772	0.72 (0.29 – 1.78)	0.474
Administration	0.65 (0.37 – 1.14)	0.136	0.55 (0.29 – 1.04)	0.065
Patient contact				
No	1 (Ref.)		1 (Ref.)	
Yes	0.58 (0.46 – 0.74)	<0.001	0.85 (0.61 – 1.18)	0.331
Working space/area				
ER	1 (Ref.)		1 (Ref.)	
ICU/isolation wards	0.84 (0.52 – 1.33)	0.452	0.94 (0.58 – 1.53)	0.804
General wards	1.07 (0.70 – 1.62)	0.762	1.11 (0.72 – 1.72)	0.630
OPD/exam rooms	1.76 (1.13 – 2.75)	0.012	1.50 (0.94 – 2.39)	0.087
RnC/PS/P	1.47 (0.82 – 2.64)	0.196	0.97 (0.51 – 1.85)	0.928
Administrative area	2.02 (1.25 – 3.27)	0.004	1.52 (0.85 – 2.73)	0.160
Others	1.51 (0.97 – 2.35)	0.067	1.20 (0.75 – 1.93)	0.452

The parameters with $p < 0.1$ in univariate were included in the multivariate analysis. ER, emergency room; ICU, intensive care unit; OPD, outpatient department; RnC/PS/P, registration and cashier/patient service/pharmacy.
